# Preventing the disaster: severe abdominal injury in child passengers of motor vehicle accidents often indicate even more serious trauma

**DOI:** 10.1007/s00068-025-02811-z

**Published:** 2025-03-20

**Authors:** Christopher Spering, R. Lefering, D. Bieler, L. Hackenberg, C. C. Dobroniak, G. Müller, W. Lehmann, Rüther H.

**Affiliations:** 1 Department of Trauma Surgery, Orthopedics and Plastic Surgery, Göttingen University Medical Center, Göttingen, Germany; 2 Institute for Research in Operative Medicine (IFOM), University of Witten / Herdecke, Cologne, Germany; 3 Department of Orthopaedics and Trauma Surgery, Heinrich Heine University Medical School, Düsseldorf, Germany; 4 Department for Trauma Surgery and Orthopaedics, Reconstructive Surgery, Hand Surgery, Burn Medicine, German Armed Forces Central Hospital Koblenz, Koblenz, Germany; 5 Chair of Automotive Engineering, Technische Universität Berlin, Berlin, Germany; 6 Committee on Emergency Medicine, Intensive Care and Trauma Management (Sektion NIS) of The German Trauma Society (DGU), Berlin, Germany

**Keywords:** Child passengers, Severely injured children, Child abdominal injury, Submarining effect, Seat belt injury, Child TBI, Child pelvic injury

## Abstract

**Purpose:**

The purpose of this study was to assess severe abdominal injury in child passengers of different ages of motor vehicle accidents and analyze the concomitant pattern of injury regarding injury severity, trauma management and outcome.

**Method:**

Data acquisition from Trauma Register DGU^®^ (TR-DGU) in a 10-years period (2010–2020) of seriously injured children (max. AIS 2+ / intensive care) 0–15 years of age, as motor vehicle passengers (cMVP) (*n* = 1,035). Primarily treated in or transferred to a German Trauma Center. Matched pairs analysis with adult severely injured motor vehicle passengers (aMVP) (age 20–50 years, *n* = 26,218), matching 1:4 (child: adult), was performed to identify causes of mortality.

**Results:**

The study group (cMVP) included 1,035 children. The mean age was 9.5 years, 50.5% were male and the mean Injury Severity Score (ISS) was 18.7 points. 93.0% were transported from scene directly to the final trauma center. Transferred patients showed a higher ISS (26 vs. 18 points), higher rate of severe traumatic brain injury (TBI), a higher rate of serious abdominal injury and a higher mortality rate (12.5% vs. 7.4%). Most of the severe abdominal injuries occurred after the third year of age (first peak between 8 and 9 years; second peak 14–15 years). Serious injuries to the pelvis show a similar distribution but less often, the same applies to thoracical injuries. Severe brain and head injuries show an antiproportional distribution to the age groups with the highest rate in the 0–1 year old (78%) and the lowest in the 14–15 year old (40%). The highest mortality rate was shown in the youngest age groups, related to TBI (AIS_TBI_ ≥ 3; 62% in 0–1 years). The matched pairs analysis shows a higher mortality rate of cMVP compared to aMVP within the first 24 h after hospital admission and a significantly higher rate of shock and unconsciousness, while the intubation rate is significantly lower.

**Conclusion:**

Child passengers of motor vehicle accidents are in need of a specific and age-related attention towards security systems. Severe injuries in children are rare, yet life threatening. The highest mortality rate is related to severe TBI, especially in the youngest children. But also severe abdominal as well as thoracic injuries their concomitant trauma need to be prevented and are indicators for even more severe injuries. It seems to be favorable for cMVP to be directly transported to designated special centers with sufficient capacity and competency to treat and manage severely injured children.

## Background

Severe abdominal blunt trauma (Abbreviated Injury Severity (AIS) ≥ 3 points) in child passengers of motor vehicle accidents (cMVP) is a rare event [1–3]. Children up to the age of 15 years only represent 3.7% of the severely injured patients who are documented in the TraumaRegister DGU^®^ (TR-DGU) [4–7]. At the same time motor vehicle accidents have been announced to be the most dangerous factor in a children’s environment for many decades, with being the leading cause of death for those under 19 years of age worldwide [8–10]. Thus, cMVP are still the leading cause of severe injury in children in developed countries [11, 12]. Yet cMVP are the main group of children who are injured (37.2%) and after pedestrians the main cause for child death in road traffic accidents (38.2%) [13].

### Child restraint systems

Nonuse and misuse of child restraint systems are common and lead to preventable severe injuries or deaths [8, 11, 14–20]. However, current knowledge about child safety seats discusses controversies related to their use especially since the morphology of the accident and the transformation of energy to the pediatric body is important to take into account [2, 8, 11, 20]. Although being restraint in a reboarded seating position, cMVP of 0–1 years of age are at the highest risk of death (in-hospital mortality rate 15.8%) [7].

### Injury pattern

According to a previous investigation in cMVP of the age 0–5 years, the 0–1 year old age group showed the significantly highest proportion of traumatic brain injuries (TBI) with Glasgow Coma Score (GCS) < 8 and severe injuries to the spine, while the 2–3-year-olds showed the significantly highest proportion of fractures especially to the lower extremity and highest proportion of cervical spine injuries of all spine injuries, and the 4–5-year-olds, the significantly highest proportion of abdominal injury and second highest proportion of cervical spine injury of all spine injuries. Severe TBI and injuries to the spine need to be prevented by age, size and body weight related passive security systems such as side- / head airbags [2, 7, 11, 15, 21]. Abdominal injuries as well as injuries to the lower spine and extremities have not been addressed with specific prevention programs so far. Both injury combinations draw special attention in literature though [3, 19, 22]: (1) abdominal injuries with a fracture of the femur (The *Submarining Effect*, caused by sliding underneath of the restraint system and cashing towards the seat in front) and (2) abdominal injuries with spinal fracture (*The Seat Belt Syndrome*, caused by the flexion injury over the seat belt). Still the age at which children should start sitting in a forward-facing position remains controversial, because injury prevention is not only related to seating position [23]. Due to its rarity, detailed reports dealing with the management of severely injured cMVP are scarce in the pediatric trauma literature. The diagnosis of severe injuries– while treating on scene or in a Trauma Resuscitation Unit (TRU)– is still challenging for trauma teams, and a high degree of awareness is necessary for rapid identification and treatment [24–27]. Passengers at the age of 0–1 year are treated with cardiopulmonary resuscitation (CPR) three times as often as older children in the prehospital setting and twice as often at admission in the TRU [7].

While older children as front-faced positioned cMVP suffer from significantly higher rates of abdominal injury [7]. Hence, the purpose of this study was to investigate the pattern of injury of cMVP throughout different ages and their specific trauma management. Therefore, the age groups were extended from 0 up to 15 years of age to also investigate if different age groups and restraint-systems might influence the severity of injury and mortality rate. In summary, the aim of the study was to evaluate severe abdominal injury in children in comparison to an adult control group with focus on the incidence, demographic characteristics, types of injuries and mortality.

## Methods

The TR-DGU of the German Trauma Society (*Deutsche Gesellschaft für Unfallchirurgie*, DGU) was founded in 1993 [27]. The aim of this multi-center database is the pseudonymized and standardized documentation of severely injured patients. Participation in TR-DGU is voluntary. For hospitals associated with the *TraumaNetzwerk DGU*^®^ (TNW) the entry of at least a basic data set is obligatory for reasons of quality assurance. Currently, approximately 30,000 cases (basic group of patients) from more than 650 hospitals are entered into the database per year. Thus for all included patients an informed consent was obtained from all subjects and/or their legal guardian.

Data are collected prospectively in four consecutive time phases from the site of the incident until discharge from hospital: (A) prehospital phase, (B) emergency/resuscitation room and initial surgery, (C) intensive care unit, and (D) discharge. Documentation includes detailed information on demographics, injury patterns, comorbidities, pre- and in-hospital management, course on intensive care unit, relevant laboratory findings including transfusion data, and outcome. Included are patients who are admitted to hospital via the resuscitation room and subsequently receive intensive or intermediate care and patients who arrive at hospital with vital signs and die before admission to the intensive care unit. The infrastructure for documentation, data management, and data analysis is provided by the Academy for Trauma Surgery (*Akademie der Unfallchirurgie GmbH*, AUC), which is affiliated with the DGU. Scientific leadership is provided by the Committee on Emergency Medicine, Intensive Care and Trauma Management (Sektion NIS) of the DGU. Participating hospitals submit their pseudonymized data to a central database via a web-based application. Scientific data analysis is approved according to a peer review procedure established by *Sektion NIS*. This study is in accordance with the publication guideline of the TR-DGU and is registered under the TR-DGU Project-ID 2022-008. All methods were carried out in accordance with the German ethics guidelines and regulations the experimental protocols including the data analysis were approved by the committee of TR-DGU and a review process prior to data acquistion.

**Fig. 1:**
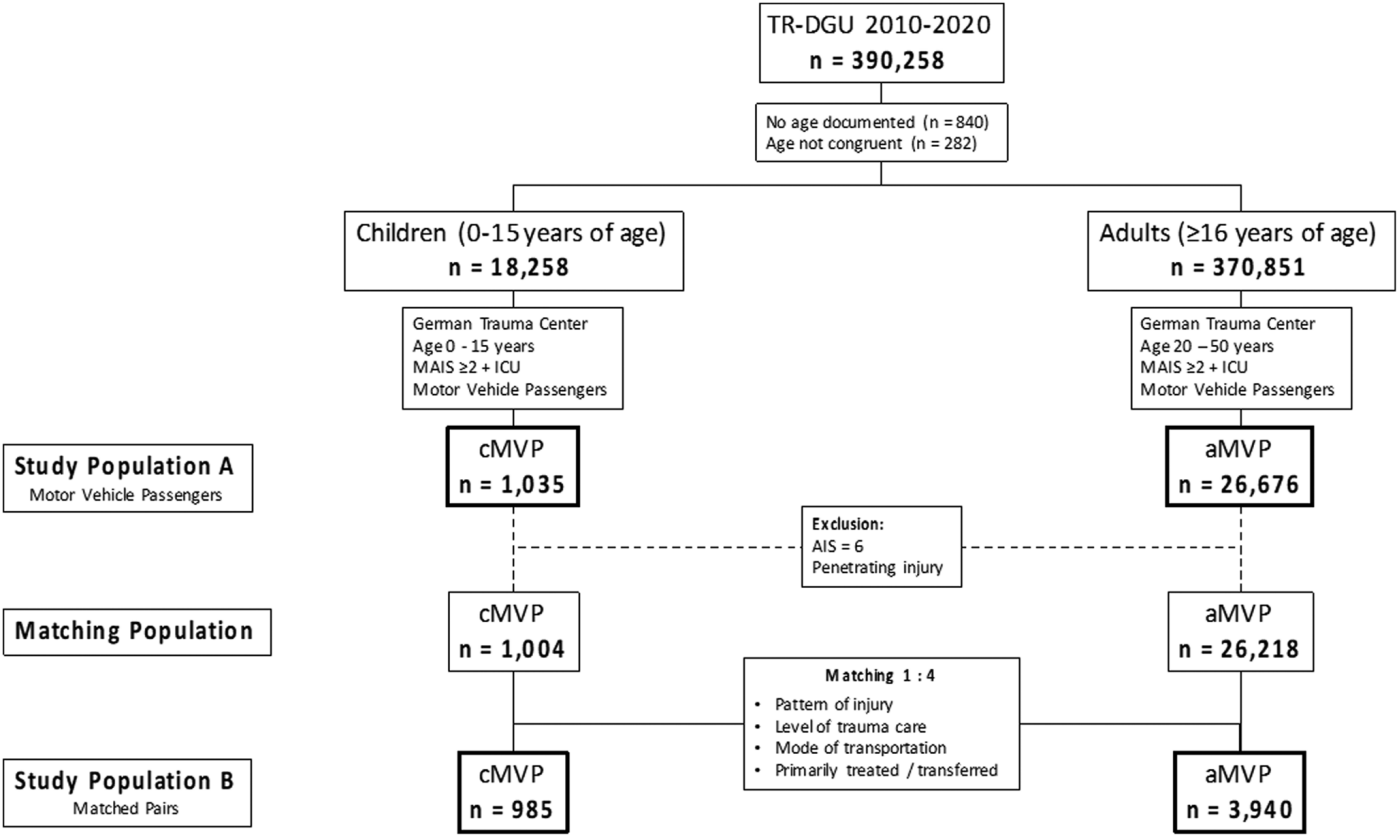
Study Population (aMVP: adult Motor Vehicle Passengers (control group); cMVP: child Motor Vehicle Passengers; AIS: Abbreviated Injury Scale; MAIS: minimum Abbreviated Injury Scale; ICU: Intensive Care Unit)

This study is a retrospective cohort study. As illustrated in Fig. 1, this study included two study populations A and B as well as a matched pairs analysis.

### Study population A

The study population A contains of children at the age of 0–15 years with a control group of adults age 20–50 years, who had been injured as motor vehicle passengers (children: cMVP, *n* = 1,035; adults: aMVP, *n* = 26,676). They were primarily admitted to a German hospital or transferred from another hospital between 2010 and 2020. According to the inclusion criteria of the TR-DGU, patients needed to suffer from a serious injury (Maximum Abbreviated Injury Scale (MAIS) ≥ 3). Patients with a MAIS severity of 2 were considered only if treated in an Intensive Care Unit (ICU), or if they died in the Trauma Resuscitation Unit (TRU). While patients under cardiopulmonary resuscitation (CPR) at admission to the trauma center were included in the dataset of TR-DGU, patients who died on scene or during transport were excluded.

In addition to descriptive analysis of different child age groups of cMVP (0–1, 2–5, 6–9, 10–13 and 14–15 years of age) and the comparison of the results of these age groups to the adult control group aMVP (20–50 years of age), a matched pairs analysis was performed, building the study population B.

### Study population B

The study population B contains of matched children and adults. Children with MAIS = 6 (*n* = 22 cMVP) as well as those with penetrating trauma (*n* = 9 cMVP) were excluded. Thus 1,004 children were matched with 26,218 aMVP (20–50 years of age). Due to a large number of adults (> 26,000) the matching was performed in a 1:4 ratio, resulting in 1 child passenger being matched to 4 adults. The matching of 1:4 ratio is preferable due to an increase of the statistical confidence level. The following criteria defined the matching:

- Injury pattern including head; thorax; abdomen; extremity with the AIS-categories 0–1 / 2–3 and 4–5 (MAIS = 6 was excluded).
- Level of trauma care (Level 1 / 2 / 3 trauma centers).
- Transportation from scene to trauma center by helicopter / ground ambulance.
- Primary admission / transfer.

In the matching process 985 children could be matched each with four randomly selected adults. In 19 cMVP cases no match was found, or less than 4 adults were available for matching. The study population B thus consists of 985 cMVP and 3,940 matched aMVP.

Statistics were performed using SPSS^®^ (Version 29, IBM Inc., Armonk, NY, USA). Descriptive analysis was done with counts and percentages for categorical variables and mean with standard deviation (SD) for continuous measurements. In case of considerably skewed data, median and inter-quartile range (IQR) were provided in addition. Significance was defined as a p-value < 0.05 using the Chi-Squared test and Mann-Whitney-U test / Kruskal-Wallis test (for 2 / 3 groups, respectively) for metric and ordinal characteristics. Outcome and prognosis parameters were calculated and put into relation to the risk of death estimation (RISC II score) [4].

## Results

### Study population (study population A)

**Fig. 2:**
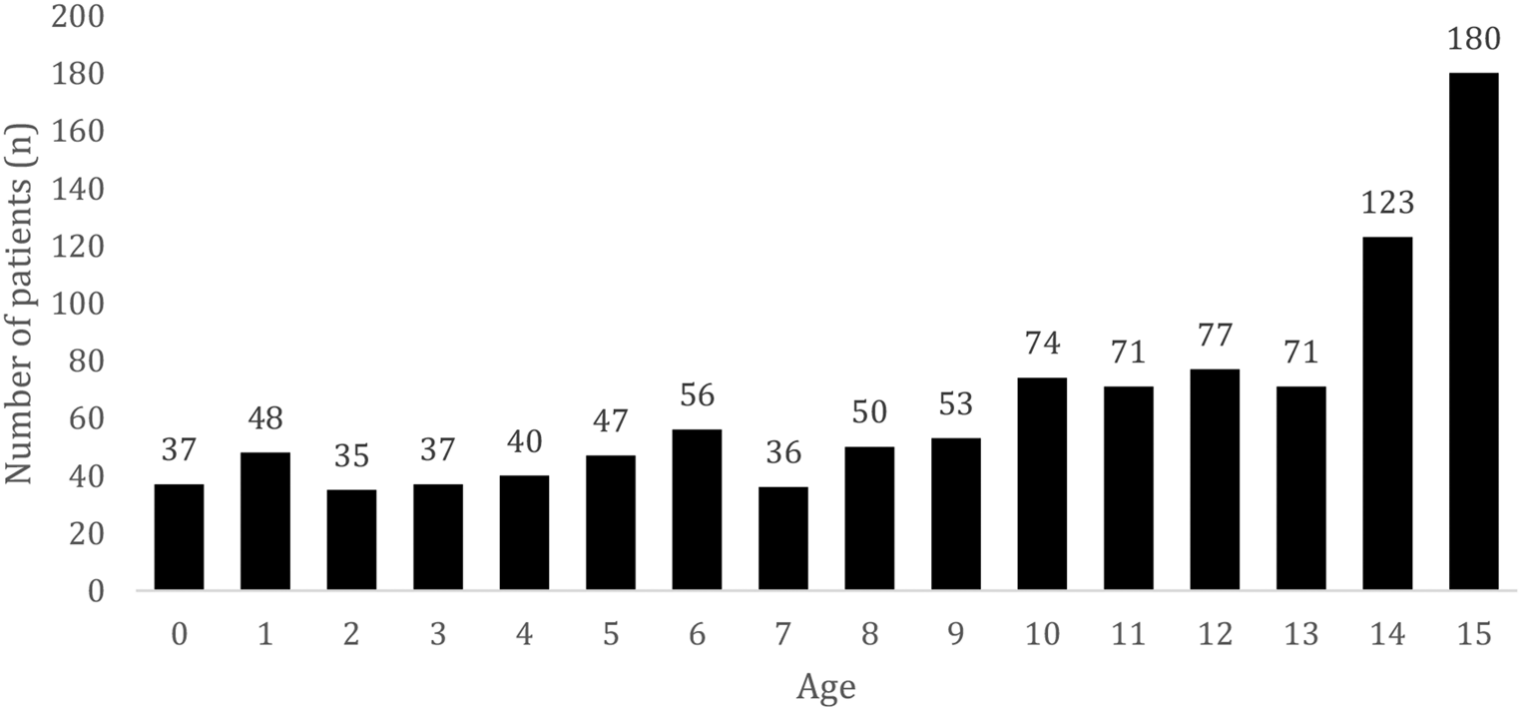
Age distribution of cMVP (*n* = 1,035)

The study group included 9,751 children (0–15 years of age), who were severely injured in road traffic accidents in a period of ten years (2010–2020) of whom 10.6% (*n* = 1,035) were injured as motor vehicle passengers (cMVP). A group of 26,676 adult (20–50 years of age) motor vehicle passengers served as control group (Fig. 1). The mean age within this study population was 9.5 years, 50.5% were male and the mean Injury Severity Score (ISS) was 18.7 points. Most of the patients (93.0%) were transported from scene directly to the treating trauma center, while 7.0% (*n* = 72) needed a transfer to another trauma center (treated in a Level 1 hospital: 71.0%, Level 2: 23.4% and Level 3: 5.6% of the children). The transferred patients showed a higher ISS (26 vs. 18), higher rate of severe TBI (AIS ≥ 3; 49% vs. 33%), a prolonged time to reach the final treating hospital (221 vs. 68 min.), a higher rate of severe abdominal injury (AIS ≥ 2; 40% vs. 28%) and a higher mortality rate (12.5% vs. 7.4%). The overall mortality rate during hospital stay was 7.0% (Table 1). As shown in Table 2; Fig. 2, almost 50% of cMVP were older than 10 years. About one third of the study population (29.3%, *n* = 303) presented a severe abdominal injury (AIS_Abdomen_ ≥ 2) with a mean age of 10.7 years, mean ISS of 22.8 points and mortality rate of 8.9% in this subgroup.

**Table 1:** Descriptive analysis of the study population A (age 0–15 years; child motor vehicle passenger (cMVP) *n* = 1,035 versus cMVP with abdominal injury (AIS ≥ 2) *n* = 303 and control (aMVP, age 20–50 years) *n* = 26,676

	cMVPn = 1,035 (100%)	Level I Trauma Center *n* = 735 (71.0%)	Level II Trauma Center *n* = 242 (23.4%)	Level III Trauma Center *n* = 58 (5.6%)	Abdominal Injury (AIS ≥ 2) *n* = 303 (29.3%)	aMVP *n* = 26,676 (100%)
Age [years] mean (SD) / median	9.5 (4.7)/11	9.2 (4.7)/10	10.2 (4.7)/12	11.8 (4.1)/13.5	10.7 (3.8)/10	33.0 (9.6)/31
Male [%] (n)	50.5 (542)	49.9 (366)	48.3 (117)	65.5 (38)	48.2 (146)	68.0 (18112)
Primary admission [%] (n)	93.0 (963)	71.0 (735)	23.4 (242)	5.6 (58)	90.4 (274)	94.3 (25149)
Transferred in from other hospital [%] (n)	7.0 (72)	9.4 (69)	1.2 (3)	0 (0)	9.6 (29)	5.7 (1527)
Injury Severity Score (ISS) [points] mean (SD) / median	18.7 (14.5)/14	20.5 (15.4)/17	15.0 (11.2)/12.5	10.6 (8.4)/9	22.8 (15.1)/18	17.9 (12.5)/14
Mortality rate [%] (n)	7.0 (72)	9.1 (67)	5.0 (12)	1.7 (1)	8.9 (27)	4.8 (1287)

**Table 2:** Age distribution of children injured as motor vehicle passengers (cMVP)

Age group [year]	Proportion of cMVP *n* = 1,035
0–1	85 (8.2%)
2–5	159 (15.4%)
6–9	195 (18.8%)
10–13	293 (28.3%)
14–15	303 (19.3%)

### Diagnostic management

**Fig. 3:**
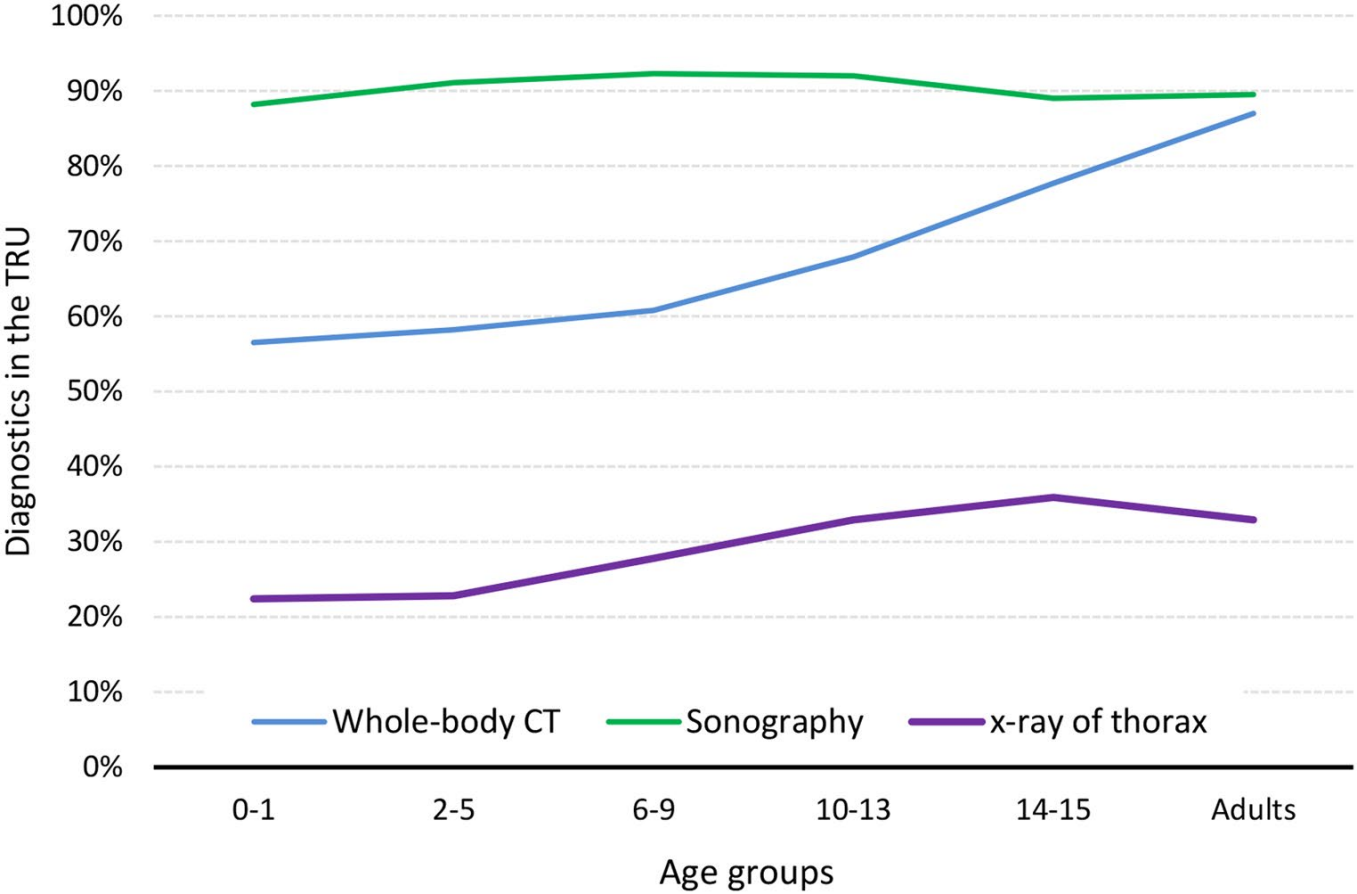
Age-group related diagnostic management in cMVP (*n* = 1,035) versus adults (control group aMVP: 20–50 years, *n* = 26,676)

The diagnostic work up when admitted to the TRU did only differ significantly between cMVP with or without severe abdominal trauma in regard of the Whole Body CT scan (WBCT). Still only 80.3% of the cMVP with severe abdominal injury received a WBCT. If selective CT scans are taken into account (i.e., cCT), cMVP without severe abdominal injury received more often selective CTscans. CMVP received significantly less often a CT scan in the TRU than aMVP. 90% received extended Focused Assessment with Sonography in Trauma (eFAST), about 30% received x-ray and about 80% underwent WBCT (Table 3). Interesting enough though, there was a significance towards the diagnostic management of adult (20–50 years of age MVP), who received WBCT more frequently than children, even though this already is the population of AIS_Abdomen_ ≥ 2 + ICU-treatment. As shown in Fig. 3, the rate of WBCT rises continuously after the age of 9 years (50–60% in the age groups 0–1, 2–5, 6–9; 70% in the age group 10–13; 80% age group 14–15 and almost 90% in adults), while sonography seems to be established in all age groups and x-ray only played a role in about 20% of the youngest cMVP and in about 30% of older cMVP and adults. MRI scan does not seem to play a standardized role in the acute diagnostic management of severely cMVP presently.

**Table 3:** Initial diagnostic management in the trauma resuscitation unit (primary admissions only) in cMVP with and without relevant abdominal injury (AIS 2+) compared to adult controls (Study population A, primary admissions only)

Diagnostic procedure	cMVP with abdominal injury *n* = 265	cMVP without abdominal injury *n* = 614	*p*-value	aMVP *n* = 25,025
eFAST	92.5%	92.0%	0.83	90.6%
X-ray	30.9%	35.8%	0.161	34.7%
Any CT	78.7%	80.3%	0.58	93.3%
WBCT	76.2%	66.4%	0.004	89.7%
MRI	0.8%	1.1%	0.73	0.2%

Abbreviations: eFAST: extended focused assessment with sonography in trauma, WBCT: whole-body computed tomography, MRI: magnetic resonance imaging

### Pattern of injury

**Fig. 4:**
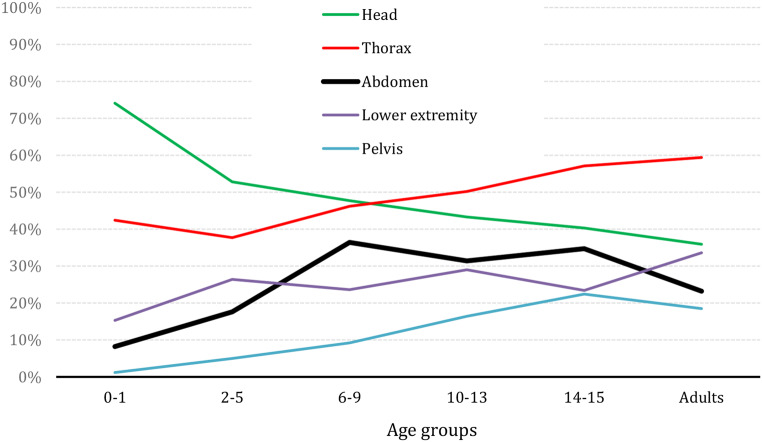
Age-group related pattern of injury of children as cMVP (*n* = 1,035). Age groups: 0–1: *n* = 85 (8.2%); 2–5: *n* = 159 (15.4%); 6–9: *n* = 195 (18.8%); 10–13: *n* = 293 (28.3%); 14–15: *n* = 303 (29.3%) and adults (control group aMVP: 20–50 years, *n* = 26,676)

About one third (29.3%, *n* = 303) of the patients showed relevant abdominal injury (AIS_Abdomen_ ≥ 2). Their mean ISS were 22.8 points compared to 18.7 points of all cMVP. The pattern and extend of concomitant injuries are shown in Table 4 with 35% head, 64% thoracic, 31% upper extremity, 22% spine, 18% lower extremity and 17% pelvic injury. In comparison to the rest of the study population (*n* = 732)– cMVP without abdominal injury– cMVP with abdominal injury showed significantly more often severe thoracic injuries and a clear tendency towards more severe injuries to the spine (AIS_Spine_ ≥ 3). Although statistically not significantly higher, injuries of the upper extremity and the pelvis were more common in children with abdominal injuries. Severe injuries to the head, face and lower extremity were seen more often in cMVP without abdominal injury with significantly more often severe TBI. As shown in Fig. 4, abdominal injury plays a minor role in the youngest age groups of cMVP, rising significantly to the age group of 6–9 years, almost reaching a plateau at around 30% before being reduced to about 20% in adults. The opposite applies to severe TBI, with the highest proportion in the youngest age group (78% in 0–1 year old), 50% in 2–5 year olds and with an ongoing decrease in the incidence by about 5 years per age group to about 35% in adults. Thoracic injuries continuously rise from about 40% in the youngest cMVP to 60% in adults. Injury to the pelvis does not play a role in the youngest age group of cMVP but reaches the highest incidence in the of group of 14–15 years of age (22%), while the lower extremity seems to be more less steadily involved between 20 and 35% with the highest incidence in the adult group of MVP.

**Table 4:** Concomitant injured body region of cMVP in the age of 0–15 years with and without severe abdominal injury. Only injuries with a severity of at least AIS 2 were considered here (Study population A)

	With Abdominal Injury *n* = 303	Without Abdominal Injury *n* = 732	*p*-value
Head	35% (105)	53% (384)	< 0.001
Face	9% (26)	14% (100)	0.023
Thorax	64% (194)	43% (312)	< 0.001
Upper extremity	31% (94)	27% (197)	0.20
Lower extremity	18% (55)	28% (202)	0.001
Pelvis	17% (52)	12% (91)	0.48
Spine	22% (67)	17% (127)	0.080

In regard to severe abdominal injuries (AIS_Abdomen_ ≥ 2) 58% of them showed liver contusions with hematoma, 36% liver lacerations, 36% contusions of the spleen with hematoma and 36% minor and 34% severe lacerations of the spleen. Small bowl contusions with perforation were found in 29% (Table 5, showing the most common abdominal injuries with *n* > 25). Most of the severe abdominal injuries occurred after the third year of age with a first peak between 8 and 9 years of age and a second peak in the 14–15 years age group. Severe injuries to the pelvis (AIS ≥ 2) show a similar distribution but less often, the same applies to thorax injuries but more often than abdominal injuries.

**Table 5:** Severity of abdominal injury in cMVP (*n* = 1,035)

AIS severity of abdominal injury	No. of children *n* = 1,035	Diagnosis (*n* > 25)
0	683	--
1	49	soft tissue injury (*n* = 65)
2	157	Liver contusion, hematoma (*n* = 58)
		Liver laceration (*n* = 36)
		Spleen contusion, hematoma (*n* = 36)
		Spleen laceration (*n* = 36)
		Kidney contusion, hematoma (*n* = 35)
3	93	Severe Spleen laceration (*n* = 34)
		Small Bowl perforation (*n* = 29)
4	42	Severe injury of the spleen (*n* = 11) and liver (*n* = 10)
5	11	Severe injury to the liver (*n* = 5)

### Outcome

**Fig. 5:**
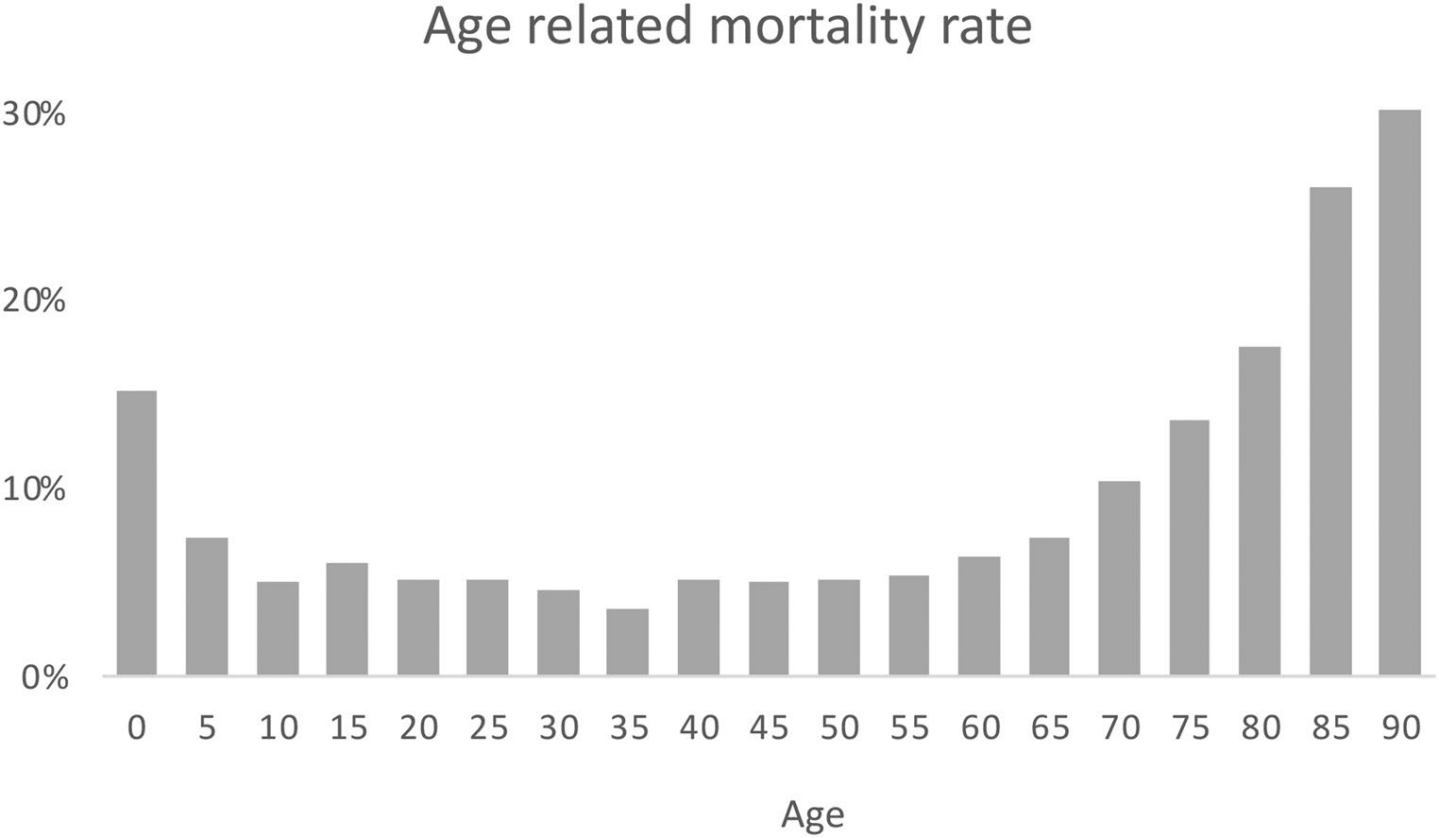
Age related mortality rate in MVP during hospital stay

**Fig. 6:**
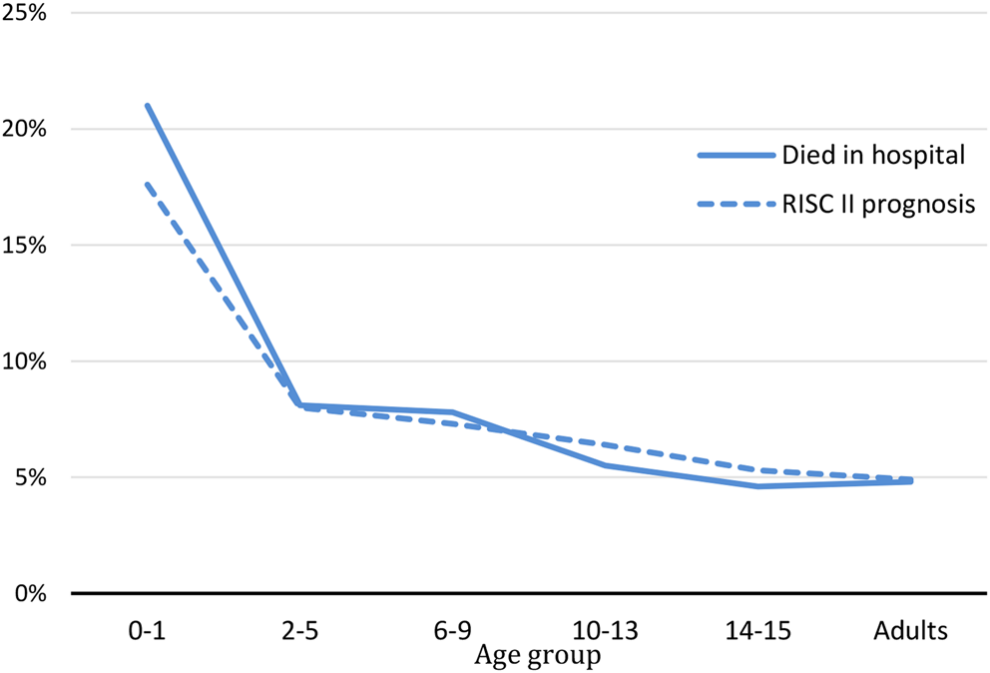
Observed (died in hospital) and expected (RISC II prognosis) mortality rate in primary admitted children (cMVP) and adults (aMVP)

The mortality rates divided into 5-years-steps show with about 20% the highest rate in the youngest age group, almost as high as the mortality rate of > 80-year old MVP. The mortality rate distribution is shown in Fig. 5. In regard to the RISC-II Score [24] the highest mortality rate was shown in the youngest age groups (22% in 0–1 years of age), diminishing significantly already to the 2–5 year old (Fig. 6). The RISC-II score shows only in the youngest age group a higher mortality rate than expected. Since no child in the age of 0–1 with abdominal trauma died, the high mortality rate in this age groups seems to be related to TBI (AIS_TBI_ ≥ 3) 62% in 0–1 years of age.

The matched pairs analysis (Table 6) shows a higher mortality rate of cMVP compared to adult MVP within the first 24 h after hospital admission (3.7% vs. 2.9%; p-value = 0.26) and a significantly higher rate of patients in shock (14.1% vs. 10.1%; p-value = 0.002) and unconsciousness (19.9% vs. 18.7%; p-value = 0.44), while the intubation rate is significantly lower in children (29.6% vs. 34.0%; p-value = 0.013). Due to a large number of adults (> 26,000) the matching was performed in a 1:4 ratio, resulting in 1 child passenger being matched to 4 adults. The proportion of patients at cardiac arrest is in cMVP higher than in their matched adults (3.2% vs. 2.1%; p-value = 0.073). The mean length of stay on ICU was 6.1 days in adult MVP versus 5.1 days in cMVP. Since the median and IQR are almost similar, there seems to be more extended treatment needs in adults on ICU (long-term patients). In regard to multiorgan failure which are not documented in all patients, there seem to be no difference in age, but in regard to sepsis the incidence was 4.2% in adults versus 2.2% in cMVP (p-value = 0.036). If treated on ICU (about 93% both groups), the proportion of ventilation was significant lower in children (36.9% vs. 42.5%; p-value = 0.003). The initial diagnostic strategy within the TRU showed significant differences in the two matched groups, not describing the diagnostics after admission to ICU though (cMVP vs. adultMVP: eFAST: 92.5% vs. 88.7%; WBCT: 67.9% vs. 89.0%; chest x-ray: 30.3% vs. 39.1%; p-value < 0.001).

**Table 6:** Matched pairs analysis. Within the matching process, 985 cMVP could be matched to 3,940 adults (Study population B). Items marked with * were the matching criteria

	Adult MVP *n* = 3,940	cMVP *n* = 985	*p*-value
Age [years]	32.6 (9.6)	9.6 (4.7)	---
Sex (male proportion)	67.9%	50.9%	< 0.001
Level of Trauma Care*1 2 3	71.1% 23.4% 5.6%	71.1% 23.4% 5.6%	---
Transferred Patients*	6.5%	6.5%	---
Injury Severity Score (ISS) (SD)	17.7 (12.0)	17.0 (11.6)	0.73
Severe TBI (AIS 4+)*	20.2%	20.2%	---
Severe abdominal trauma (AIS 4+)	4.5%	4.5%	---
Abdominal trauma (AIS 2+)*	32.8%	32.8%	---
Only primarily treated patients	*n* = 3,684	*n* = 921	
Prehospital treatment:			
Intubation	34.0%	29.6%	0.013
Catecholamine application	8.4%	7.1%	0.39
Thoracic drain	4.2%	2.6%	0.14
Cardiac arrest / CPR	2.1%	3.2%	0.073
Unconsciousness (GCS 3–8)	18.7%	19.9%	0.44
Shock (BP ≤ 90 mmHg)	10.1%	14.1%	0.002
Time from accident to admission (min) (SD)	70 (29)	70 (28)	0.19
Trauma Resuscitation Unit:			
Sonography (eFAST)	88.7%	92.5%	< 0.001
Whole-Body-CT	89.0%	67.9%	< 0.001
Chest x-ray	39.1%	30.3%	< 0.001
Admission to Intensive Care	93.9%	93.5%	0.65
Ventilated (if on ICU)	42.5%	36.9%	0.003
All Patients			
Mortality rate within 24 h	2.9%	3.7%	0.26
Hospital mortality rate	5.4%	5.6%	0.81
Length of stay on ICU [days] mean (median) [IQR]	6.1 (2) [1–6]	5.1 (2) [1–5]	0.058
Length of stay in hospital [days] median [IQR]	11 [6–21]	9 [4–16]	< 0.001

## Discussion

In this paper, we provide data showing that children and especially the youngest age group still is in danger when it comes to car passengers of motor vehicle accidents. It seems odd, that in a country with relatively high-quality standards for the car production and safety development especially regarding mobility, children still remain in danger of suffering severe injuries or death. As we discussed in Spering et al. (2022), it is not the reboarding that saves live, but a variety of different factors that need to be considered when it comes to injury prevention in cMVPs [7].

Still, the epidemiology of the dataset shows not only a high number of cMVP in regard to all children in the age of 0–14 years being injured in traffic accidents but also the need of initial treatment in dedicated Level 1 Trauma Centers. Even though only based on 7% severely injured children within 10 years who were secondary transferred, but the higher injury severity, higher TBI rate, more severe single injuries and higher mortality rates in the transferred patients cannot only be explained by the longer time of transport. A recent evaluation of secondary transferred patients within the trauma network of Germany emphasizes the need of (1) admission to Level 1 Trauma Centers for special groups of patients (e.g. pediatric patients) and (2) the need of continuous re-assessment and aftercare of these severely injured [5]. We need a sensibility for these patients already prior to hospital admission, even if the initial clinical exam might appear normal, to adequately address the danger of severe injury. Huecker et al. (2023) just recently showed the relevance of continuous examination after high velocity accidents for child car passengers [28]. This is especially true, if abdominal injuries are suspected or diagnosed on scene (i.e., “seat belt sign”– contusions, petechiae and band-like pattern of abrasions across the lower abdomen). The underlying data show, that children with abdominal and thoracic injuries (AIS ≥ 3) caused by blunt trauma might have equivocal or absent physical findings. Thus, the WBCT seems to be used less often than in adults. But since the analysis of included children shows injuries with an AIS ≥ 3, a CT scan or MRI must have been performed at least after admission to ICU. With these findings and the fact that inadequate physical functioning after abdominal and thoracic injuries might develop several days after the car crash, repeated physical examination and radiological re-evaluation need to be performed. The presented data provide a hint, that pelvic, abdominal, and thoracic injuries could be indicators for even more severe injuries e.g., TBI or spinal injuries. Sweed et al. (2020) showed in their case reports, that rare injuries, e.g., abdominal aortic injury, can easily be missed initially [29]. Although abdominal aortic trauma was associated with duodenal injuries in almost all described cases it still seems to be a rare event and was not found in the present dataset of the TR-DGU. In concordance to Szadkowski et al. (2017) and Huecker et al. (2023) repeated physical examinations are mandatory as physiological or clinical signs can be missed while treatment on scene or during admission at the TRU [28, 30]. Furthermore, a diagnosis of hollow viscus injury in children is difficult even with CT scan, and only a close clinical follow-up can help identify children with these injuries [31–34]. However, we might keep in mind that CT is useful and specific in solid organ injury diagnosis but lacks sensitivity when diagnosing bowel and mesenteric injury [24]. The exploratory laparotomy results described in this paper are with a good agreement with Breen et al. (1997) [35]. Their assessment of the diagnostic performance of CT signs in blunt abdominal and mesenteric injury were that bowel wall thickening, bowel wall discontinuity, extraluminal air, and mesenteric hematoma are all reasonably specific (84%, 95%, 100%, 94%) but not sensitive (50%, 58%, 44%, 54%). They also reported that the presence of moderate to large volume of intraperitoneal fluid without visible organ damage is an important sign [35]. To conclude, reassessment of the physical examination, laboratory parameters, and at least sonography as a diagnostic reassessment, should be implemented in critical patients and in those in whom an occult injury is suspected [25, 34, 35].

Although the present data of the TR-DGU cannot provide information about seat belt and child seat use, failure to use or the misuse of restraints poses a much greater risk for children, than might be expected. According to Durbin et al. (2023) car seat use reduces the risk for death to infants (0–1 year of age) by 71% [36] while booster seat use reduces the risk for serious injury by 45% for children aged 4 to 8 years when compared with seatbelt use alone [37]. For older children and adolescents, 3-point seatbelt use reduces the risk for death and serious injury by approximately half [38]. Even when car seats and booster seats are implemented, improper installation rates are very high, with an estimated combined international rate of misuse at about 46% [38]. The so called “submarining effect” describes the injury pattern of abdominal injury in addition to a femur shaft fracture caused by sliding of the body below the restraining system. Thus, the seating position and the restraining system needs to be re-evaluated and pattern of injury as well as age related changes of anatomical condition need to be considered if severely injured cMVP are investigated closely. The aim is to be more efficient in preventing severe injuries in children by using medical data from the TR-DGU.

Independently from the use, misuse or drivers’ behavior and experience [23], the data of this publication ought to rethink not only the safety systems of modern cars, but also our medical trauma and emergency approach towards the early management of cMVP. The matched pairs analysis shows differences in the management of adult MVP to child MVP in regard to a higher mortality rate of cMVP compared to adult MVP within the first 24 h after hospital admission (3.7% vs. 2.9%; p-value = 0.26) and a significantly higher rate of patients in shock (14.1% vs. 10.1%; p-value = 0.002) and unconsciousness (19.9% vs. 18.7%; p-value = 0.44), while the intubation rate is significantly lower in children (29.6% vs. 34.0%; p-value = 0.013). The proportion of patients at cardiac arrest is in cMVP higher than in their matched adults (3.2% vs. 2.1%; p-value = 0.073). The mean length of stay on ICU was 6.1 days in adult MVP versus 5.1 days in cMVP. This descriptive analysis provides a hint towards more prospective management of children. It could be the case, that children compensate in the early hours to days after accident so that trauma teams do not detect the underlying injury and do not perform further diagnostics until children show obvious decompensation. That could be one of the reasons for the higher mortality rate. Also, the interpretation of unconsciousness might be wrong, or trauma teams are not routinely trained in child intubation. The higher proportion of cardiac arrest though, should warrant a rethinking of our initial treatment and whether it is progressive enough.

Related to abdominal injury, it is not only the seating position and the medical work up but also the sensibility of undetectable injuries. In children with a seatbelt sign and abdominal pain or tenderness on examination, an abdominal CT scan should be strongly considered given the high risk of intraabdominal injury. Abdominal ultrasound alone and peritoneal lavage are not recommended because of their lack of specificity.

The dearth of large prospective trials makes it difficult to create concrete management guidelines for children presenting with a seatbelt sign but no initial abdominal tenderness or pain. The finding that approximately 2% of these cases require surgical intervention and the present data showing mostly minor abdominal injury (AIS ≤ 3) suggests that these patients should be admitted for observation and have serial abdominal examinations to detect evolving physical signs suggestive of intraabdominal injury [39].

Given the extremely high sensitivity of newer CT scanners, discharge home with specific follow-up instructions may be considered for children with a seatbelt sign who have a normal abdominal CT scan and no abdominal symptoms [40]. If the CT scan is negative for injury but abdominal pain persists, continued observation with serial examinations is indicated, although no clear guidelines on length of observation currently exist.

## Conclusion

Child passengers of motor vehicle accidents need a specific and age-related attention towards security systems. Severe injuries in children are rare, yet life threatening. The highest mortality rate is related to severe TBI, especially in the youngest children. However, also severe abdominal and thoracic injuries and their concomitant trauma need to be prevented and are indicators for more severe injuries. Appropriate use of child safety seats and booster seats with 3-point restraints significantly reduce, but do not eliminate, the risk for intra-abdominal, spinal and brain injuries in pediatric patients. Early diagnosis of small bowel injury may be difficult and requires a high index of suspicion to facilitate appropriate diagnostic testing and consultation. These incidents emphasize the required serious awareness of the complete spectrum of intra-abdominal injuries in restrained pediatric passengers in motor vehicle crashes. This awareness might lead to a rigorous investigation of possible injuries, even when external signs and physical indexes seem to be normal.

### Limitations

The data of the TR-DGU though does not provide information about the seating position and even if the percentage of children who are seated in a reboarding position in the ages of 0–1 years is almost 100%, there might still be a minimal percentage of children who are positioned incorrectly even in that age group.

Due to the fact, that the data inclusion criteria to the TR-DGU only addresses children who got admitted to the trauma center, we do not have enough information about the children who died during the prehospital trauma management. Though this number seems to be quite low, according the Destatis-report (2021) [13].

The difference in diagnostic management between children and adult multiple trauma patients can only be interpreted carefully due to the fact that CT or MRI scans which were performed after admission to the ICU are not documented. The documentation of MRI-scans as part of the early trauma management was not part of the TR-DGU before the year 2015.

Due to age related physiological circumstances, a lower blood pressure (< 90 mmHg) in the youngest age groups, if not age adjusted, can lead to a misinterpretation in regard to shock which is only detectable in the TR-DGU dataset in concordance to a matched age of the patient.

The focus of this study is the evaluation of severely injured children. We did not investigate children with minor or no injury as MVP. Thus, the presented data cannot provide information about the efficiency of child safety systems in general. The fact, that severe injury in the presented age groups is seldom might be an effect of a general high quality of safety systems, if applied correctly.

## Data Availability

Data is provided within the manuscript.
